# Validation of Arabic versions of the child psychosocial distress screener and pediatric symptom checklist for young adolescents living in vulnerable communities in Lebanon

**DOI:** 10.1186/s13033-024-00640-y

**Published:** 2024-05-30

**Authors:** Felicity L. Brown, Frederik Steen, Karine Taha, Gabriela V. Koppenol-Gonzalez, May Aoun, Richard Bryant, Mark J D. Jordans

**Affiliations:** 1 Research and Development Department, War Child Alliance, Helmholtzstraat 61G, Amsterdam, The Netherlands; 2 Amsterdam Institute of Social Science Research, University of Amsterdam, Amsterdam , 1098LE The Netherlands; 3 War Child Alliance Lebanon, Beirut, Lebanon; 4 School of Psychology, University of New South Wales, Sydney, NSW Australia

**Keywords:** Child psychosocial distress screener, Pediatric symptom checklist, Psychosocial screening, Lebanon, Validation, Adolescence

## Abstract

**Background:**

In humanitarian settings, brief screening instruments for child psychological distress have potential to assist in assessing prevalence, monitoring outcomes, and identifying children and adolescents in most need of scarce resources, given few mental health professionals for diagnostic services. Yet, there are few validated screening tools available, particularly in Arabic.

**Methods:**

We translated and adapted the Child Psychosocial Distress Screener (CPDS) and the Pediatric Symptom Checklist (PSC) and conducted a validation study with 85 adolescents (aged 10–15) in Lebanon. We assessed internal consistency; test-retest reliability; convergent validity between adolescent- and caregiver-report and between the two measures; ability to distinguish between clinical and non-clinical samples; and concurrent validity against psychiatrist interview using the Kiddie Schedule for Affective Disorders and Schizophrenia.

**Results:**

The translated and adapted child-reported PSC-17 and PSC-35, and caregiver-reported PSC-35 all showed adequate internal consistency and test-retest reliability and high concurrent validity with psychiatrist interview and were able to distinguish between clinical and non-clinical samples. However, the caregiver-reported PSC-17 did not demonstrate adequate performance in this setting. Child-reported versions of the PSC outperformed caregiver-reported versions and the 35-item PSC scales showed stronger performance than 17-item scales. The CPDS showed adequate convergent validity with the PSC, ability to distinguish between clinical and non-clinical samples, and concurrent validity with psychiatrist interview. Internal consistency was low for the CPDS, likely due to the nature of the brief risk-screening tool. There were discrepancies between caregiver and child-reports, worthy of future investigation. For indication of any diagnosis requiring treatment, we recommend cut-offs of 5 for CPDS, 12 for child-reported PSC-17, 21 for child-reported PSC-35, and 26 for caregiver-reported PSC-35.

**Conclusions:**

The Arabic PSC and CPDS are reliable and valid instruments for use as primary screening tools in Lebanon. Further research is needed to understand discrepancies between adolescent and caregiver reports, and optimal methods of using multiple informants.

## Background

Global rates of forced displacement due to armed conflict are at unprecedented levels [1]. Children’s exposure to armed conflict leads to increased risk of psychological distress and disorders [2]. There is increasing focus on the importance of providing mental health care to children and adolescents in these contexts, however significant barriers to service provision exist, including the availability of mental health specialists to assist in assessment, diagnosis, and treatment [3]. In order to adequately understand rates of psychological distress, accurate identification of those needing treatment, and the effectiveness of services, there is a need for adequate self-report measurement tools that can be administered by non-professionals.

The majority of validated psychological assessment tools are developed in high income countries. While there is increasing use of these tools in different cultural and linguistic populations, their cultural and contextual validity is often not adequately considered [4, 5]. In humanitarian contexts, assessing the validity of tools is particularly important, for several reasons: (i) the presence of diverse and complex situational stressors; (ii) varying cultural backgrounds of communities; (iii) increased vulnerability for psychological distress coupled with limited service availability, making accurate identification of those needing treatment vital; and (iv) the potential for non-adapted tools to inflate rates of clinical disorders, or to miss other valid complaints. Furthermore, many existing tools are diagnosis-specific, meaning that multiple tools are required to detect treatment needs across the spectrum of possible disorders. This is infeasible in low resource settings, where broad measures of distress may be better fit to capture the range of complaints requiring treatment [6]. As there is a growing move towards transdiagnostic interventions that are applicable to a range of symptoms of distress and can be delivered by non-specialists [6], there is a need for accompanying broad screening tools.

A large number of refugees globally are Arabic speaking, with 6.7 million refugees from Syria since the onset of the civil war constituting the largest refugee group in 2020 [1]. Translating measures into Arabic poses particular challenges. Most available translations use written modern standard Arabic, which can diverge from dialects spoken in different regions, leading to issues for standardization, validity, and reliability of measures. Since the majority of research involving Arabic-speaking people relies on self-report measures developed and normed internationally, it is essential that careful considerations are incorporated in decisions around translation, adaptation, and norming, to ensure methodological validity and reliability of implementation [5, 7].

In this study we aimed to evaluate the psychometric properties of two translated and adapted psychological distress screeners in Lebanon- the Child Psychosocial Distress Screener (CPDS) [8] and adolescent- and caregiver-report 17- and 35- item versions of the Pediatric Symptom Checklist (PSC) [9].

## Methods

### Design

**Fig. 1:**
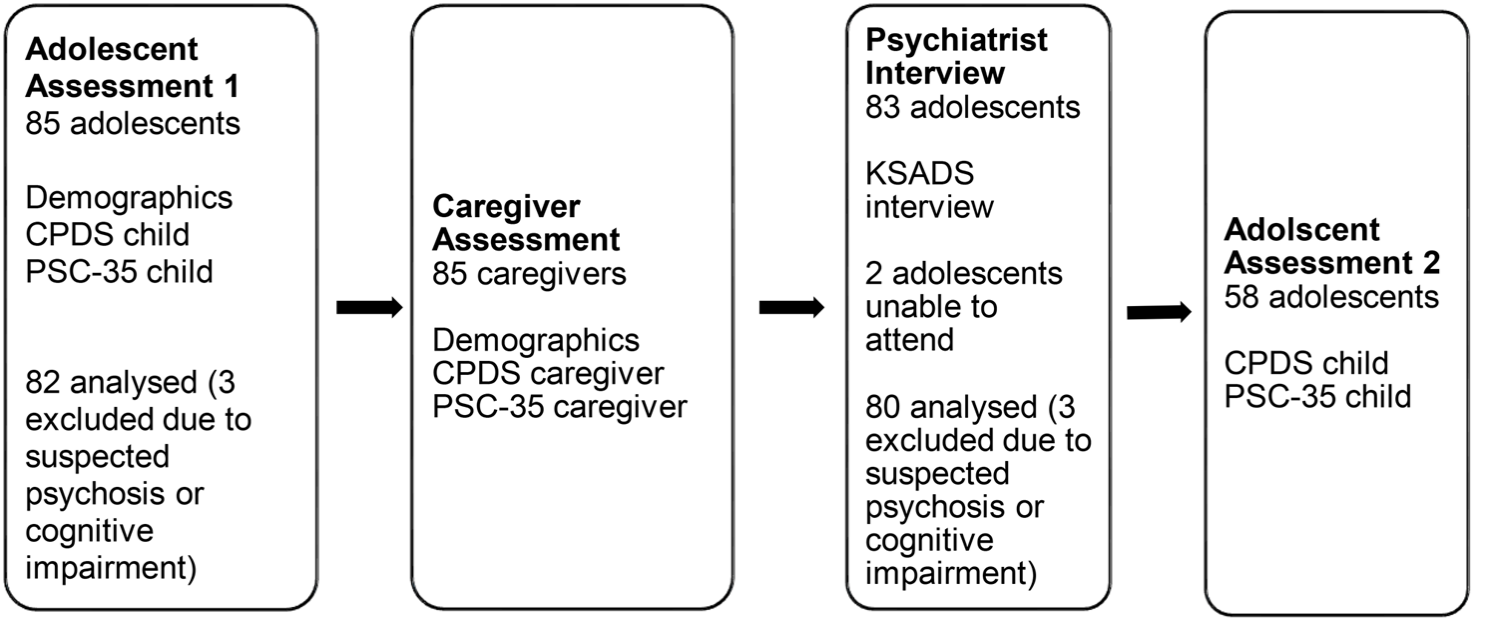
Design of validation study for Pediatric Symptom Checklist and Child Psychosocial Distress Screener for adolescents in Lebanon

We systematically translated and adapted the tools and administered them with 85 adolescents (age 10–15) and their caregivers, followed by a structured clinical interview conducted by a psychiatrist (*n* = 83). A sub-sample (*n* = 58) of adolescents repeated the two measures approximately 10 days later, with no intervention provided in between. First, we assessed internal consistency and test-retest reliabilities. Second, we assessed convergent validity between adolescent and caregiver report and between the CPDS and PSC. Third, we examined known-groups validity by testing the ability of the measures to distinguish between clinical and non-clinical groups of adolescents. Finally, we assessed concurrent validity by comparing the scores on the measures with a *‘gold standard’* semi-structured clinical interview for psychiatric disorder. We were interested in three types of *‘caseness’*: (i) whether the psychiatrist detected any diagnosis, (ii) whether the psychiatrist indicated that the adolescent needed treatment, and (iii) both diagnosis and need for treatment. The study design is depicted in Fig. 1 below. Ethical approval was obtained from St Joseph’s University Beirut (USJ-2017-24).

### Setting

Lebanon is a middle-income, Arabic-speaking country that has experienced prolonged internal and external conflicts and hosts the highest number of refugees per capita globally [1]. We conducted our study via three community-based organisations partnering with War Child Alliance, in Chatila Palestinian camp in Beirut, and Mina and Beb el Ramel in Tripoli.

### Recruitment, sample size, inclusion criteria

Participants were adolescents aged 10 to 15 years of Lebanese, Syrian, Palestinian, or Egyptian nationality. We did not select adolescents based on clinical status but aimed to achieve a representative sample of those attending services. Recruitment was conducted via regular outreach approaches, which target adolescents living in areas of high adversity and vulnerability. Adolescents displaying significant cognitive disability or psychosis via psychiatrist assessments were excluded from analysis, to avoid compromising reliability of the self-report findings.

### Measures

#### Pediatric symptom checklist

The PSC comprises 35 symptoms (including internalising, externalising, somatic, social, and academic difficulties) rated for frequency of occurrence on a three-point scale from 0 (never) to 2 (often) [9]. The total score ranges from 0 to 70. The PSC-35 has shown high internal consistency, test-retest reliability, and strong agreement, specificity and sensitivity compared to validated measures or clinician assessments [10]. For children aged 6–16 years the standard cut-off score on the caregiver-report tool is 28 or above; the PSC does not provide a diagnosis, but instead indicates emotional and behavioural problems that may warrant further clinician assessment. It has demonstrated feasibility and sustainability as a primary screening tool; however, developers stress the need to determine valid cut-off scores for new populations [9]. Less research exists on child-report versions; one study found that PSC child-report significantly correlated with caregiver and teacher report of child dysfunction, and with child-reported symptoms of depression and anxiety on other measures [11]. Of those identified as needing follow-up on the child-report, 71% had not been identified using the caregiver-reported PSC, highlighting the importance of youth-report measures. The recommended cut-off from this study in a low-income population in the USA is 30.

A shorter 17-item checklist, consisting of a sub-set of items from the longer scale, has been developed and the caregiver-report version has shown high agreement with clinician diagnoses, performing as well as existing child-reported screeners, with the exception of identifying anxiety disorders [12]. Recommended cut-offs for child and caregiver-report versions are 15 and above. One validation study conducted in Turkey found strong internal consistency, test-retest reliability, and discriminant and concurrent validity when compared with scores on the Child Behaviour Checklist [13], but found a cut-off of 12 to be optimal.

We translated and adapted the PSC to Arabic for use in Lebanon, following a systematic process based on best practice [14]. This involved: (i) forward and back translation to modern standard Arabic by independent translators; (ii) translation workshop with bilingual Lebanese professionals in Lebanon; (iii) forward and back translation to simple spoken Arabic (considered suitable for Syrian and Lebanese populations) by independent translators; (iv) translation workshop with bilingual Lebanese professionals in Lebanon; (v) cognitive interviewing with target Arabic-speaking adolescents in Lebanon, including Syrian refugees; (vi) a translation workshop with bilingual Lebanese team members to review necessary translation changes needed; vi) pilot testing through a pre-post study of an educational intervention in Lebanon; (vii) further cognitive interviewing; (viii) a further translation workshop to further refine the translations; (ix) final adjustments to translation of the items; (x) back translation by a bilingual psychologist not involved in prior steps; xi) final agreement by two bilingual psychologists. Items between the 17- and 35-item versions are identical, and adolescent- and caregiver-reported items only differ on whether they refer to ‘you’ or ‘your child’, therefore any changes made on one version of the measure were also reflected in other versions.

The main changes were:

1. Item 1 (*complains of aches and pains*) and Item 3 (*tires easily, has little energy*)- were switched in order, as starting with a somatic symptom was confusing for adolescents.
2. Item 7- the idiom *‘like driven by a motor’* was not applicable and replaced by *‘feels like she/he can’t stop moving’*.
3. Items 5 and 6- references to teachers and schools were expanded to include other adults and other daily activities, to be more applicable to out-of-school adolescents.
4. Item 13- the concept of *‘hopeless’* is not easily understood in Arabic, and this question was reframed and reversed to *‘thinks that the coming days will be better’*.
5. Items 29 (*does not listen to rules*) and Item 31 (*does not understand other people’s feelings*)- were reversed, as the negative framing was complex to understand in Arabic.

#### Child Psychosocial distress screener

The CPDS was developed in the context of a psychosocial program for children in four conflict-affected countries [15]. It is a primary screening tool for children aged 8–14 years, that assesses psychosocial distress, as opposed to specific disorders, promoting early detection of children in need for psychosocial care. The tool consists of 5 child-reported items, and 2 caregiver-reported items, with general questions which are then elucidated using probes developed for the specific context; for example the first question ‘*Did you experience any aversive events?*’, is then followed by locally relevant examples. Higher scores indicate more psychosocial distress. The CPDS has robust cross-cultural construct validity [4] and has been validated in Burundi samples, with an optimal cut-off of 8, with diagnostic sensitivity between 0.84 and 0.94 and specificity between 0.60 and 0.75 [7]. In Lebanon, we developed probes directly from findings of a rapid qualitative assessment [16], using the Arabic words used by respondents. The general questions were translated initially following forward and back translation to simple spoken Arabic (considered suitable for Syrian and Lebanese populations) by independent translators, followed by a translation workshop with bilingual professionals. The questions and the probes were then refined via the following iterative steps (i) cognitive interviewing with target adolescents (Syrian and Lebanese); (ii) a translation workshop to review necessary translation changes needed; (iii) further cognitive interviewing; (iv) a further translation workshop to further refine the translations; (v) final adjustments to translation of the items; (vi) back translation by a bilingual psychologist not involved in prior steps; (vii) final agreement by two bilingual psychologists.

#### Kiddie schedule for affective disorders and schizophrenia

The Kiddie Schedule for Affective Disorders and Schizophrenia (KSADS) [17] is a comprehensive semi-structured clinical interview designed to identify mental disorders in children according to Diagnostic and Statistical Manual of Mental Disorders Fourth Edition [18] classifications. The instrument was translated to Arabic by Un Ponte Per in Iraq. Three psychiatrists in Lebanon received three half-day Skype training sessions with the tool developers at STAR Center, University of Pittsburgh, and regular supervision via four Skype sessions. During the training sessions, they reviewed terminology and determined adjustments that may be needed for local dialects.

Assessments started with an introduction to build rapport and obtain biographical information. The KSADS interview was conducted, including assessment of nonspecific distress, functioning, coping and support mechanisms, and contextual information. Additional information was obtained from caregivers when required. Psychiatrists recorded the presence of current diagnoses (definite, probable, or partial remission), whether participant data should be excluded due to psychosis or cognitive disability, and whether the adolescent required treatment. We focused only on current symptoms, rather than lifetime diagnoses. We assessed for substance use disorders but did not code tobacco use disorder as a diagnostic category for analysis. We omitted assessment of enuresis, encopresis, mania, anorexia, bulimia, conduct disorder, and tic disorder. This was determined based on cross-cultural relevance, and the extent to which these would be captured on self-report.

#### Procedure

The procedure is illustrated in Fig. 1. First, caregivers provided written informed consent for their participation and their adolescents’, then adolescents provided assent. The CPDS and then the PSC were administered via a one-to-one interview with a trained research assistant who read the questions, provided respondents with pictorial Likert scales to aid responding, and recorded responses in Kobo data collection software (Adolescent Assessment 1 and Caregiver Assessment).

Adolescents were subsequently interviewed by a psychiatrist on a separate day within two weeks of the first assessment (Psychiatrist Interview). Psychiatrists were blind to questionnaire results. Two adolescents were unable to attend interviews due to scheduling difficulties. Psychiatrists recorded diagnosis/es (if any), and a dichotomous response on treatment indication (yes/no). Case-consensus meetings were held with the three psychiatrists for nine interviews, whereby one psychiatrist presented their case findings in detail, and the other two psychiatrists provided blind opinions on the cases on diagnoses and treatment indication.

Approximately 14 days after assessment 1 (*M* = 14.4 days, range = 10–17 days), and after the psychiatrist interview, a subsample of adolescents took part in a follow up assessment (Adolescent Assessment 2). Convenience sampling was used, with all adolescents invited to an assessment day, and interviews conducted with those attending.

No financial incentives were provided for participating. A small refreshment was provided to adolescents and caregivers at each assessment, and a small reimbursement was provided to cover transportation costs.

Adolescents requiring urgent treatment were referred following national referral pathways and organizational procedures. Eligible adolescents were also invited to participate in a pilot of a new psychological intervention.

### Statistical analysis

#### Preliminary analyses and psychometric properties

From Adolescent Assessment T1, Caregiver Assessment, and Psychiatrist interviews, we present demographics data and diagnostic and treatment status descriptively. To assess internal consistency of the PSC and the CPDS, we calculated Cronbach’s alpha for each measure. Confirmatory factor analysis was not considered to be valid given the small sample size and properties of the dataset [19].

#### Reliability and validity analyses

We examined convergent validity via Pearson correlations between PSC child, CPDS child, PSC caregiver, CPDS caregiver, and CPDS total scores. We calculated test-retest reliability for 58 cases using Intraclass Correlation Coefficients (ICC). We considered a coefficient below 0.40 poor, 0.40 to 0.59 fair, 0.60 to.74 good; and 0.75 to 1.00 excellent [20]. We conducted between-group *t*-tests to compare PSC and CPDS mean scores between groups indicated for treatment or not, and between groups with any diagnosis versus none.

We calculated receiver operating characteristic (ROC) curves and examined area under the curve (AUC) for:

1. Overall accuracy of CPDS to distinguish caseness.
2. Overall accuracy of PSC-35 child- and caregiver-report to distinguish caseness.
3. Overall accuracy of PSC-17 child- and caregiver-report to distinguish caseness.

We considered caseness in multiple ways.

1. Any diagnosis included in the KSADs.
2. Treatment indication.
3. Any diagnosis plus treatment indication.
4. Internalizing diagnosis (removing ADHD and ODD).
5. Internalizing diagnosis plus treatment indication.

For each measure we also calculated positive predictive value (PPV; proportion of positive test results for true caseness) negative predictive value (NPV; proportion of negative test results for true non-caseness), optimal cut-offs, and sensitivity and specificity.

#### Handling missing data

We noted a very small proportion of missing data, with the exception of school-related items which were not relevant for many out-of-school adolescents. Of all the items at T1 there were 9 items with 1 to 5 missing values (1.2 to 6.1%) and one item with 14 missing values (17.1%). Analyses were conducted using replacement with the proportion score of the answered items and multiple imputation with three data sets. Findings did not differ significantly; therefore, multiple imputation analyses are reported.

## Results

### Participants

Eighty-five adolescents completed the initial assessment with their caregivers. Eighty-three adolescents completed assessments with psychiatrists. We excluded three participants from analyses, one due to likely psychotic symptoms, one due to likely significant cognitive impairment, and one due to both. Thirty-three adolescents were assessed as having a diagnosis (probable, definite, or in partial remission). Eighteen were indicated as needing psychiatric or psychological treatment. Demographics and diagnoses are shown in Table 1 below.

**Table 1:** Demographics of adolescent sample in lebanon

		*N*	%
Sex	Female	50	61
	Male	32	39
Age	10	20	24
	11	22	27
	12	19	23
	13	12	15
	14	7	9
	15	2	2
Nationality	Lebanese	12	15
	Syrian	67	82
	Palestinian	2	2
	Egyptian	1	1
Attends school	Yes	30	37
	No	52	63
Generates income	Yes	6	7
	No	76	93
Responding caregiver	Mother	73	89
	Father	7	9
	Other	2	2
Diagnosis	Major depressive disorder	12	15
	Dysthymia	3	4
	Adjustment disorder (depression)	3	4
	Adjustment disorder (anxiety)	2	2
	Panic disorder	1	1
	Separation anxiety disorder	18	22
	Avoidant disorder of childhood	2	2
	Simple phobia	8	10
	Social phobia	8	10
	Agoraphobia	1	1
	Overanxious	5	6
	Generalised anxiety disorder	7	9
	Obsessive compulsive disorder	1	1
	Post-traumatic stress disorder	4	5
	Acute stress disorder	1	1
	Attention deficit hyperactivity disorder	4	5
	Oppositional defiant disorder	2	2

The sample consisted predominantly of Syrian refugees, slightly more females than males, and mostly adolescents not attending school. The most common diagnoses were major depressive disorder (15%) and separation anxiety disorder (22%), followed by simple phobias (10%), social phobia (10%), and generalized anxiety disorder (9%).

As shown in Table 2, the PSC-35 child and caregiver versions both had good internal consistency, and the PSC-17 child and caregiver versions had adequate internal consistency. Internal consistency for the CPDS was unacceptable, but this scale consists of only five adolescent-reported items plus two caregiver-reported item, and is designed to indicate treatment need, rather than assess one construct. Adolescent-reported PSC and CPDS scores correlated highly, as did caregiver-reported PSC and CPDS scores, but adolescent and caregiver reports did not correlate with each other. Test-retest reliability had fair to good clinical significance for adolescent measures [20].

**Table 2:** Psychometric properties, correlations, and test-retest reliability of PSC and CPDS

	Cronbach’s alpha	Test-retest reliability (ICC)	PSC-35 Child	PSC-17 Child	PSC-35 Caregiver	PSC-17 Caregiver	CPDS Child	CPDS Caregiver	CPDS Total
PSC-35 Child	0.80	0.69 (good)			0.18		0.56**	0.19	0.59**
PSC-17 Child	0.61	0.58 (fair)				0.09	0.46**	0.18	0.50**
PSC-35 Caregiver	0.80	n/a					− 0.08	0.47**	0.14
PSC-17 Caregiver	0.72	n/a					− 0.11	0.36**	0.06
CPDS Child	0.66	0.63 (good)						− 0.04	0.90**
CPDS Caregiver	n/a	n/a							0.41**
CPDS Total	0.49	n/a							

*Note* PSC, Pediatric Symptom Checklist; CPDS, Child Psychosocial Distress Screener; ICC, Intraclass Correlation Coefficient

** *p* < .01

There was a significant difference in scores on the CPDS, adolescent-reported PSC-35, adolescent-reported PSC-17, and caregiver-reported PSC-35 for adolescents indicated for treatment versus those not indicated for treatment, and for adolescents assessed as having a diagnosis versus not having a diagnosis (See Table 3). There was no significant difference between groups on caregiver-reported PSC-17.

**Table 3:** Results of between group t-tests on PSC and CPDS scores for children identified as “Cases” or “Non-Cases”

	Treatment Indicated?			Diagnosis?		
	Case (*M*)	Non-Case (*M*)	*t*-test	Case (*M*)	Non-Case (*M*)	*t*-test
PSC-35 Child	25.56	14.67	*t* (21.6)= -4.83, *p* < .001	22.30	13.48	*t* (78)= -5.60, *p* < .001
PSC-17 Child	13.39	8.10	*t* (78)= -5.61, *p* < .001	11.76	7.57	*t* (78)= -5.12, *p* < .001
PSC-35 Caregiver	24.85	19.47	*t* (78)=-2.30, *p* < .05	23.27	18.86	*t* (78)=-2.21, *p* < .05
PSC-17 Caregiver	13.65	12.00	*t* (57)=-1.01, *p* = .30	12.93	12.00	*t* (57)=-0.66, *p* = .51
CPDS	6.11	3.76	*t* (78)= -3.58, *p* < .001	5.76	3.26	*t* (78)= -4,73, *p* < .001

*Note* PSC, Pediatric Symptom Checklist; CPDS, Child Psychosocial Distress Screener

As shown in Tables 4 and 5, the optimal cut-off for the PSC measures varied depending on the criterion for ‘caseness’ and whether adolescent-reported or caregiver-reported. For CPDS, a cut-off of 5 was found to be optimal. It should be noted that a cut-off of 4 had higher sensitivity (.82), however specificity was reduced at this cutoff and overall accuracy was lower. The CPDS had adequate AUC. AUCs were higher for adolescent-reported PSC-17 (.80-.83) and PSC-35 (.83-.85) than caregiver PSC-17 (.55-.61) and PSC-35 (.65-.73) and were higher for 35-item scales than 17-item scales.

**Table 4:** Area under the curve and predictive properties for optimal cut-off scores for PSC and CPDS

	KSADS- any diagnosis							KSADS- treatment indicated					KSADS- diagnosis and treatment indicated					
Measure	AUC	Cut off^a^	Sens	Spec	PPV	NPV	AUC	Cut off^a^	Sens	Spec	PPV	NPV	AUC	Cut off^a^	Sens	Spec	PPV	NPV
PSC-35 Child	0.83	17	0.82	0.70	0.66	0.85	0.83	16	0.83	0.55	0.35	0.92	0.85	21	0.71	0.86	0.58	0.92
PSC-17 Child	0.80	10	0.61	0.72	0.61	0.72	0.83	12	0.67	0.90	0.67	0.90	0.83	12	0.71	0.91	0.67	0.92
PSC-35 Caregiver	0.65	19	0.78	0.51	0.53	0.77	0.71	21	0.78	0.61	0.37	0.90	0.73	26	0.65	0.81	0.48	0.90
PSC-17 Caregiver	0.55	11	0.63	0.41	0.53	0.52	0.61	12	0.65	0.55	0.37	0.79	0.61	12	0.63	0.54	0.33	0.79
CPDS	0.76	5	0.62	0.79	0.67	0.75	0.75	5	0.69	0.71	0.41	0.89	0.77	5	0.73	0.71	0.41	0.91

*Note* KSADS, Kiddie Schedule for Affective Disorders and Schizophrenia; PSC, Pediatric Symptom Checklist; CPDS, Child Psychosocial Distress Screener; AUC, Area under the curve; Sens, Sensitivity; Spec, Specificity; PPV, Positive Predictive Value; NPV, Negative Predictive Value

^a^optimal cut-off determined through examining Receiver Operating Curve Tabs

**Table 5:** Area under the curve for internalizing diagnoses only and predictive properties for optimal cut-off scores for PSC and CPDS

KSADS any internalising diagnosis							KSADS any internalising diagnosis and treatment indicated					
Measure	AUC	Cut off^a^	Sens	Spec	PPV	NPV	AUC	Cut off^a^	Sens	Spec	PPV	NPV
PSC-35 Child	0.83	17	0.82	0.70	0.66	0.85	0.85	21	0.71	0.86	0.58	0.92
PSC-17 Child	0.80	10	0.61	0.72	0.61	0.72	0.83	12	0.71	0.91	0.67	0.92
PSC-35 Caregiver	0.65	19	0.78	0.51	0.53	0.77	0.73	21	0.82	0.61	0.37	0.93
PSC-17 Caregiver	0.55	11	0.63	0.41	0.53	0.52	0.61	12	0.63	0.54	0.33	0.79
CPDS	0.76	5	0.62	0.79	0.67	0.75	0.77	5	0.73	0.71	0.41	0.91

*Note* KSADS, Kiddie Schedule for Affective Disorders and Schizophrenia; PSC, Pediatric Symptom Checklist; CPDS, Child Psychosocial Distress Screener; AUC, Area under the curve; Sens, Sensitivity; Spec, Specificity; PPV, Positive Predictive Value; NPV, Negative Predictive Value

^a^optimal cut-off determined through examining Receiver Operating Curve Tabs

## Discussion

We found that the translated and culturally adapted versions of the CPDS, adolescent-reported PSC-17 and PSC-35, and caregiver-reported PSC-35 scales have sound psychometric properties and criterion validity when delivered by non-specialists for adolescents aged 10–15 years living in Lebanon. They showed adequate test-retest reliability, ability to distinguish between clinical and non-clinical samples, and high concurrent validity compared to psychiatrist assessment. The caregiver-reported PSC-17 did not demonstrate adequate performance in this population, and both the 17- and 35-item adolescent-reported PSCs outperformed caregiver-reported versions. Similarly, 35-item PSCs showed stronger performance than shorter 17-item scales. While the PSC scales showed adequate internal consistency, the CPDS did not.

The CPDS may have had lower internal consistency due to the fact that it incorporates contextual challenges into one measure of ‘psychosocial risk’, rather than measuring diagnostic symptoms as one underlying ‘construct’. Furthermore, it is a very brief multi-source instrument, with some items reported by child and some by caregiver, which is likely to impact internal consistency. Given that the benefit of the screening tool is the brief nature and contextual focus, the low internal consistency considered alongside sound validity, may not indicate poor performance for the intended purpose.

To identify adolescents in need of treatment, we recommend a total cut-off score of 5 on the CPDS. For PSC measures, the cut-offs vary depending on the respondent, and the desired criterion. We recommend a cut-off of 12 on the adolescent-reported PSC-17, as the optimal cut-off for an adolescent needing psychological treatment, an adolescent indicated as having any diagnosis and needing treatment, and an adolescent indicated as having an internalising diagnosis and needing treatment. We recommend a cut-off of 21 on the adolescent-reported PSC-35, as the optimal cut-off for an adolescent indicated as having any diagnosis and needing treatment, and an adolescent indicated as having an internalising diagnosis and needing treatment. For caregiver-reported PSC-35, we recommend a cut-off of 21, as indicating an adolescent needing treatment, and an adolescent having an internalizing diagnosis and needing treatment. While sensitivity and specificity at these cut-offs was considered optimal, false positives for treatment indication may be elevated (indicated by the low PPVs) when using these tools. They should be used as a first step to indicate further assessment, rather than being considered as diagnostic tools. Furthermore, uncorrected prevalence rates based on these tools may over-estimate treatment need.

These cut-offs are lower than generic cut-offs recommended, (15 for PSC-17 and 28 for PSC-35 caregiver-reports) but match the cut-off for the PSC-17 identified in a Turkish sample [13]. Stoppelbein and colleagues [21] similarly found a PSC-17 cut-off of 12 in a sample of youth in the USA, with systematically different response patterns between Caucasian versus African American youth, possibly due to cultural norms in responding. Our lower cut-off score may be due to under-reporting of symptoms on an assessment, given that stigma related to mental health concerns is a widely acknowledged problem in this region [22]. It is possible that psychiatrists were able to elicit more disclosure of symptoms through interviews. Nonetheless, our experience highlights the importance of identifying culturally and contextually relevant norms to prevent over- or under-identification.

In our sample, adolescent-reported and caregiver-reported scales did not correlate. Caregiver-reported scales demonstrated lower concurrent validity with psychiatrist interviews, possibly since interviews were conducted solely with adolescents. Similar findings have been found in previous research using the PSC [23] and other scales (24–25) and discrepancies between caregiver and adolescent reports may predict future adolescent internalizing symptoms and functioning (24–25). In this study caregiver-reported questionnaires had particularly low AUC for internalizing disorders, possibly indicating that caregivers have less knowledge of internalizing symptoms as compared to externalizing symptoms. Future research will be important to further understand the reasons and implications for discrepancies in Lebanon.

In this sample, 40% of adolescents were assessed by psychiatrists as meeting criteria for at least one diagnosis, and 22% were considered to be in need of mental health treatment. A recent meta-analysis found that approximately 20% of individuals living in conflict-affected areas meet diagnostic criteria for a common mental disorder at any given time [2]. The high prevalence of diagnosable disorders found among adolescents of mixed nationalities in this study, though a small and non-representative sample, are in line with extremely high rates of psychological symptoms reported among Syrian refugees [26].

Our findings support the importance of incorporating generic screening measures for children and adolescents affected by armed conflict and adversity, beyond just trauma-related symptoms, and providing evidence-based psychological treatments that address the diverse challenges experienced. In our study, the majority of diagnoses were not trauma-focused, but rather mood and anxiety focused. While our study was not designed to determine prevalence rates, diagnoses of post-traumatic stress disorder were substantially lower than those found in epidemiological studies [26], suggesting the need for further research to understand the application of such diagnostic categories in diverse settings. While direct exposure to war-related trauma undoubtedly increases risks for a range of psychological symptoms, trauma-focused models neglect a range of important etiological factors including pervasive daily stressors, adverse family events and community environments, and do not address the broad spectrum of clinical presentations that are likely to arise [27]. Further, the use of non-specific distress screeners has been advocated in other settings, to identify broadly defined distress [28].

Our results indicate the utility of these translated and adapted tools as primary screeners with adolescents in Lebanon. The translated instruments overcome issues with other tools that commonly use modern standard Arabic, which can be misunderstood by respondents, or may be converted non-systematically to spoken dialect by assessors as they are delivering it, both of which compromise reliability and validity.

One limitation of our study was the relatively small sample size (*n* = 80). While comparable to similar studies (*n* = 65) [8, 29], more cases would have increased statistical power. Future research should conduct confirmatory factor analyses and other more advanced analyses using larger samples [5]. Additionally, test-retest assessments were conducted with a sub-sample of adolescents available on second assessment day, and we did not follow up further with those who did not attend. Therefore, our re-test sample may not have been representative of the full sample. We could not ensure that the same assessor completed the second assessment with adolescents, which may have added variance in our test-retest analyses. Additionally, adolescents participating in the study were already engaged in services, and therefore may have felt more comfortable disclosing problems, or may not have been representative of a general community sample. It will be important for future research to explore the use of these tools in different settings, including during a first contact with children and adolescents, to ensure generalizability of findings.

## Conclusions

In low resource settings, a huge mental health treatment gap exists, largely owing to unavailability of professionals to assess and identify those needing treatment, and to provide those treatments. Furthermore, there is a dearth of culturally validated screening instruments. Our study indicates the feasibility of conducting screening by non-professionals in Lebanon, using short, culturally-adapted instruments, making early detection of adolescents needing psychological treatment possible. This enables actors to: (i) identify the scope of mental health needs in a population; (ii) identify adolescents most in need of the limited services available; and (iii) adequately measure the effectiveness of these services. The use of a transdiagnostic measure of distress, rather than a narrow measure of a particular diagnosis, provides a more flexible and practical approach in a setting where mental health needs are diverse and complex.

## Data Availability

The datasets used and/or analysed during the current study are available from the corresponding author on reasonable request.

## Abbreviations

CPDS: Child Psychosocial Distress Screener
PSC: Pediatric Symptom Checklist
K-SADS: Kiddie Schedule for Affective Disorders and Schizophrenia
ICC: Intraclass Correlation Coefficients
ROC: Receiver Operating Characteristic
AUC: Area Under the Curve
